# Modeling Filamentous Cyanobacteria Reveals the Advantages of Long and Fast Trichomes for Optimizing Light Exposure

**DOI:** 10.1371/journal.pone.0022084

**Published:** 2011-07-18

**Authors:** Carlos Tamulonis, Marten Postma, Jaap Kaandorp

**Affiliations:** 1 Informatics Institute, University of Amsterdam, Amsterdam, The Netherlands; 2 Swammerdam Institute for Life Sciences, University of Amsterdam, Amsterdam, The Netherlands; University of Illinois at Urbana-Champaign, United States of America

## Abstract

Cyanobacteria form a very large and diverse phylum of prokaryotes that perform oxygenic photosynthesis. Many species of cyanobacteria live colonially in long trichomes of hundreds to thousands of cells. Of the filamentous species, many are also motile, gliding along their long axis, and display photomovement, by which a trichome modulates its gliding according to the incident light. The latter has been found to play an important role in guiding the trichomes to optimal lighting conditions, which can either inhibit the cells if the incident light is too weak, or damage the cells if too strong. We have developed a computational model for gliding filamentous photophobic cyanobacteria that allows us to perform simulations on the scale of a Petri dish using over 10^5^ individual trichomes. Using the model, we quantify the effectiveness of one commonly observed photomovement strategy—photophobic responses—in distributing large populations of trichomes optimally over a light field. The model predicts that the typical observed length and gliding speeds of filamentous cyanobacteria are optimal for the photophobic strategy. Therefore, our results suggest that not just photomovement but also the trichome shape itself improves the ability of the cyanobacteria to optimize their light exposure.

## Introduction

Cyanobacteria are a very large and diverse phylum of photoautotrophic prokaryotes [1]. They are defined by their unique combination of pigments and their ability to perform oxygenic photosynthesis. They often live in colonial aggregates that can take on a multitude of forms [2]. Of particular interest are the filamentous species, which often dominate the upper layers of microbial mats found in extreme environments such as hot springs, hypersaline water, deserts and the polar regions [3], but are also widely distributed in more mundane environments as well.

Many species of cyanobacteria are capable of gliding. Gliding is a form of cell movement that differs from crawling or swimming in that it does not rely on any obvious external organ or change in cell shape and it occurs only in the presence of a substrate [4], [5]. Gliding in filamentous cyanobacteria appears to be powered by a “slime jet” mechanism, in which the cells extrude a gel that expands quickly as it hydrates providing a propulsion force [6], [7], although some unicellular cyanobacteria use type IV pili for gliding [8]. Individual cells in a trichome have two sets of pores for extruding slime. Each set is organized in a ring at the cell septae and extrudes slime at an acute angle [9]. The sets extrude slime in opposite directions and so only one set is likely to be activated during gliding. An alternative hypothesis is that the cells use contractive elements that produce undulations running over the surface inside the slime tube like an earthworm [10]. It is further interesting to note that the trichomes rotate in a spiral fashion, the angle of which corresponds with the pitch angle of Castenholz's contractile trichomes.

The cells appear to coordinate their gliding direction by an electrical potential that establishes polarity in the trichomes, and thus establishes a “head” and the “tail” [11]. Trichomes usually reverse their polarity randomly with an average period on the order of minutes to hours [12], [13]. Many species also form a semi-rigid sheath that is left behind as a hollow tube as the trichome moves forward. When the trichome reverses direction, it can move back into the sheath or break out [14].

Cyanobacteria have strict light requirements. Too little light can result in insufficient energy production, and in some species may cause the cells to resort to heterotrophic respiration [3]. Too much light can inhibit the cells, decrease photosynthesis efficiency and cause damage by bleaching. UV radiation is especially deadly for cyanobacteria, with normal solar levels being significantly detrimental for these microorganisms in some cases [15], [16].

Filamentous cyanobacteria that live in microbial mats often migrate vertically and horizontally within the mat in order to find an optimal niche that balances their light requirements for photosynthesis against their sensitivity to photodamage. For example, the filamentous cyanobacteria *Oscillatoria* sp. and *Spirulina subsalsa* found in the hypersaline benthic mats of Guerrero Negro, Mexico migrate downwards into the lower layers during the day in order to escape the intense sunlight and then rise to the surface at dusk [17]. In contrast, the population of *Microcoleus chthonoplastes* found in hypersaline mats at Salins-de-Giraud, Camargue, France migrate to the upper layer of the mat during the day and are spread homogenously through the mat at night [18]. An *in vitro* experiment using *P. uncinatum* also demonstrated this species' tendency to migrate in order to avoid damaging radiation [15], [16]. These migrations are usually the result of some sort of photomovement, although other forms of taxis can also play a role [19].

Photomovement – the modulation of cell movement as a function of the incident light – is employed by the cyanoabacteria as a means to find optimal light conditions in their environment. There are three types of photomovement: photokinesis, phototaxis and photophobic responses [12], [20], [21].

Photokinetic microorganisms modulate their gliding speed according to the incident light intensity. For example, the speed with which *Phormidium autumnale* glides increases linearly with the incident light intensity [22].

Phototactic microorganisms move according to the direction of the light within the environment, such that positively phototactic species will tend to move roughly parallel to the light and towards the light source. Species such as *Phormidium uncinatum* cannot steer directly towards the light, but rely on random collisions to orient themselves in the right direction, after which they tend to move more towards the light source. Others, such as *Anabaena variabilis*, can steer by bending the trichome [23].

Finally, photophobic microorganisms respond to spatial and temporal light gradients. A step-up photophobic reaction occurs when an organism enters a brighter area field from a darker one and then reverses direction, thus avoiding the bright light. The opposite reaction, called a step-down reaction, occurs when an organism enters a dark area from a bright area and then reverses direction, thus remaining in the light.

Gliding filamentous cyanobacteria, such as *Phormidium uncinatum*, display step-down photophobic reactions when their ‘heads’ (i.e. the leading 10–20% of the trichome) are shaded, causing the trichome to reverse direction. Conversely, *P. uncinatum* also reacts to light incident on the trichome tail [11]. Light incident on the middle of the trichome has no effect. Shining a narrow beam of light on the rear of a trichome will induce it to reverse direction. The trichomes appear to compare the incident light between the head and the tail and if the tail receives more light than the head the trichome is highly likely to reverse its direction [13]. Although considered a form of phototaxis by Gabai [13], the response does not depend on the direction of the light but on a light intensity gradient, and thus may better be considered a step-up photophobic response. However, the tail step-up response seems to have a different physiological basis than the step-down reactions originating from the head [13], which are triggered through the photosynthetic apparatus [24].

The effects of photomovement on a population of trichomes can be observed in vitro by shining a column of light, called a light trap, on a culture of *P. uncinatum*. The light scattered by the trichomes and particles already in the trap spot draws the trichomes in the surrounding dark area towards the illuminated area by positive phototaxis. The trichomes enter the trap from the darker area freely, but will reverse direction whenever moving from the lighter area to the darker area due to step-down photophobic responses. Over the course of a few hours the trichomes will completely fill the trap, marking it with a darker colour [25], [26], [27].

**Figure 1:**
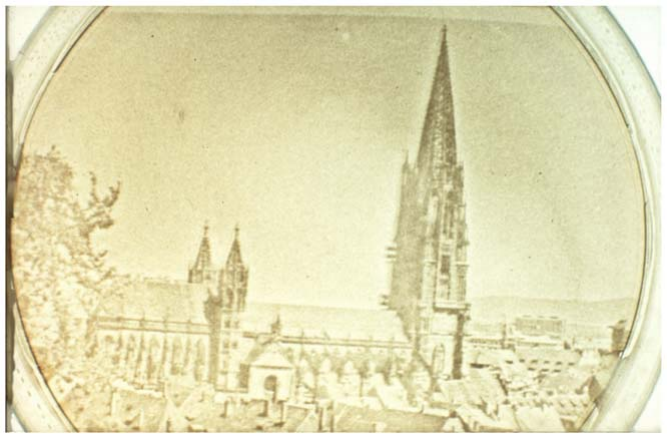
Häder's cyanograph experiment. A photographic negative is projected onto a Petri dish containing a culture of photophobic filamentous cyanobacteria (*Phormidium uncinatum*). The trichomes cover the lighter areas of the projection while uncovering the darker areas producing a photographic positive. Petri dish is 10 cm wide. Courtesy of Donat-Peter Häder.

Häder demonstrated that trichomes can position themselves quite precisely within their environment through photomovement. In Häder's cyanograph experiment [12], [28], a photographic negative is projected onto a Petri dish containing a culture of *P. uncinatum*. After a few hours, the trichomes move away from the darker areas onto the lighter areas, forming a photographic positive on the culture (Figure 1). The experiment demonstrates that photomovement is effective not just for discrete light traps, but for minutely patterned, continuously differentiated light fields as well.

In the study presented here, we explore the advantages of photophobia as a means for optimizing light exposure through the use of a computational model for motile trichomes. Each trichome is modeled as a self-propelled long thin elastic rod equipped with a simple model of photophobia, including both step-up and step-down reactions. Previous models of the photomovement of filamentous cyanobacteria by Burkart [26] and Häder [27] using partial differential equations demonstrated how a combination of phototaxis and step-down photophobic responses can lead to the accumulation of trichomes in light traps. Our model is a large individual based system that allows us to simulate responses to static and dynamic light fields as well as quantify the advantage of using the photophobic mechanism for optimizing light exposure compared to random gliding and also show how the length and gliding speed of the trichomes can influence this process.

Somewhat similar models exist of gliding bacteria applied to Myxobacteria, whose fruiting-body formation behavior is used as a model system for primitive morphogenesis. Myxobacteria not only glide using a slime extrusion (A-motility) but also by extension and retraction of type IV pili that can attach to other individuals and pull them closer (S-motility). Like the cyanobacteria, they also reverse their direction of movement periodically. Wu et al. [29], [30] developed a cell-based model of the gliding *M. xanthus* and predicted that the periodic reversals are essential for swarming and increase cell alignment. They were also able to predict the optimum reversal period for swarm expansion to within measurement error of actual cells. Models similar to the one used in this study have recently been used to study the dynamics of cell-cell collisions in Myxobacteria [31], [32]. Peruani et al. [33] also use a similar deterministic model for myxobacteria, albeit for short inflexible rods and only on a small-scale. The advantage of our approach is that we can scale our simulations to the full domain size (i.e. a 10 cm Petri dish) by using massively parallel graphics processing units (GPUs).

## Methods

### Model of filamentous cyanobacteria

We have used an ad-hoc two-dimensional computational model for gliding phototactic trichomes. Each trichome is represented by a chain of connected edges, which in turn is a sequence of vertices. The vertices are spaced such that each edge is approximately *l* = 50 µm long, depending on the total length (*L*) of the trichome. The movement of the trichomes is based on simple Newtonian mechanics and the position of each vertex, **r**, is governed by:
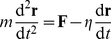
(1)where *t* denotes time, **F** denotes the total force acting on a vertex, *m* and *η* denote the mass and the damping parameter respectively. For all vertices we take *m* = 1 and *η* = 1, chosen so that the dynamics of the system are highly damped (see Discussion for a critical assessment of the methods).

All the phenomena in the model are described in terms of vector forces. Running a simulation step consists of calculating the sum of all the forces acting on each vertex and solving the resulting system of differential equations using the Velocity Verlet numerical integration method with timestep *Δt*, typically 0.1 s. The boundary conditions are such that the vertices are restricted to a circular domain with an inelastic boundary. Any force or impulse against the domain boundary is met with an equal reaction force and so the following conditions are enforced after each time step
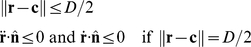
(2)where **c**  =  <*D*/2, *D*/2> denotes the centre of the circular domain and 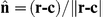 is the unitary vector pointing from the centre of the domain towards the vertex.

For each time step all the forces exerted on each vertex *i* are calculated. The forces arise from (a) trichome elasticity, including tensile and bending strain, (b) gliding and (c) collisions:
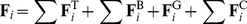
(3)


#### Trichome elasticity

**Figure 2:**
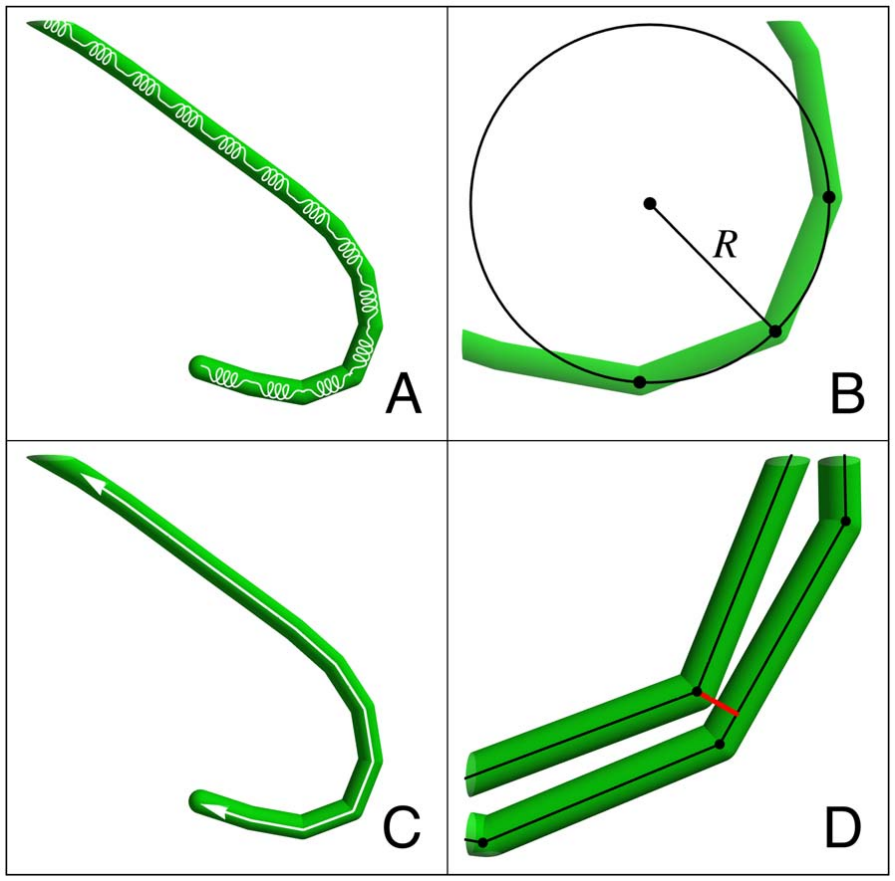
Model components. (A) Trichomes are modeled as thin flexible rods that are discretized into sequences of 50 µm edges. Each edge is loaded with a linear spring. (B) The local bending moment is a function of the radius of curvature. (C) Trichomes can glide along their long axis and reverse their direction of movement photophobically. (D) Trichome collisions are defined between edge-vertex pairs. A vertex that penetrates an edge's volume is repulsed by equal and opposite forces between the pair.

Each trichome is modeled as a thin flexible rod with diameter *d* and total length *L*. Each trichome is discretized into *N*  =  round(*L*/50) segments of equal length and yielding *N* + 1 equally spaced vertices <**r**_0_, …, **r** *_N_*> that represent each trichome (Figure 2A). Each vertex is subject to a force that derives from the local tensile and bending strain at the vertex. Torsion is ignored.

The force between any two vertices <**r** *_i_*, **r** *_i_*_+1_> due to tensile strain is given by:
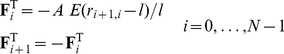
(4)where 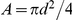 denotes the rod's cross sectional area, *E* denotes the elastic modulus of the rod, 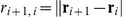 denotes the distance between the two vertices *i* and *i+1*, 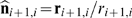 denotes the unitary vector pointing from 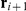 to 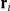 and *l* denotes the length of the segments.

For the bending resistance forces we use a phenomenological model based on the concept that the bending resistance of a straight elastic rod is directly proportional to the curvature of the rod [34]:
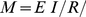
(5)where *M* denotes the bending moment generated at the ends of the rod, *E* denotes the elastic modulus of the rod, *I* = π/4 × (*d*/2)^4^ denotes the second moment of area of the rod's cross section the and *R* denotes the radius of curvature (Figure 2B). We use this model to calculate the local bending forces within the trichome by applying it to pairs of consecutive segments. The bending moment generated by two connected segments composed of three vertices 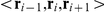 is estimated by assuming that the radius of curvature is the radius of the unique circle defined by the three vertices (Figure 2B). The bending moment is converted into forces acting on the three vertices by assuming that the two outer vertices rotate around the middle vertex, each with moment *M*, such that the total torque and total force acting on the three vertices is zero:
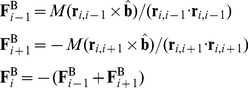
(6)where 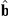 denotes a pseudo-vector normal to the plane defined by the three vertices:
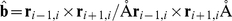
(7)and is oriented into or out of the plane according to whether the trichome is bent to the “left” or to the “right” at the middle vertex. The resulting set of forces tends to move the three vertices onto a straight line, while the tensile forces restore each segment to its initial length.

#### Gliding motility

As described in the introduction, most filamentous cyanobacteria glide along their long axis. They are unable to steer (with the notable exception of *Anabaena* sp.), but they do reverse their direction of movement randomly [14]. The propulsion force is probably generated by slime extruding from pores located all along the trichome's length [6]. The slime swells as it leaves the pores and generates a force tangential to the trichome. Likewise, the virtual trichomes are assumed to be able to exert a tangential force against a substrate. The propulsion force is applied at each vertex and is tangential to the trichome at that point (Figure 2C). The trichomes are also assumed to be polarized and that this polarization controls the direction of movement of the trichomes, resulting in the following equation:
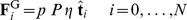
(8)where *p* = {−1, 1} denotes the polarity of the trichome, *P* denotes the propulsion speed and 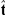 denotes a vector tangent to the trichome at vertex *i* given by:
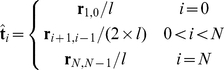
(9)


#### Collisions

Each trichome has its own exclusive volume that is impenetrable. Each trichome segment is modeled as a capsule-shaped volume composed of a cylinder with semi-spherical caps. Any vertex that enters an edge's exclusive area is repelled (Figure 2D). The repulsion force intensity is given by:
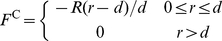
(10)where *r* denotes the length of the displacement vector between the edge and the vertex, *d* denotes the trichome diameter and *R* the repulsion strength.

The direction of the force is along the direction of the displacement vector, which is the shortest vector connecting the edge to the vertex. The displacement vector **d** between an edge **e**  =  **r** *_i+1_* **– r** *_i_*, and a vertex **r** *_k_* is given by:
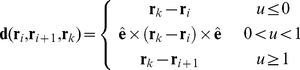
(11)where 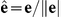 denotes the normalized edge vector and
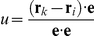
is the normalized projection of the vertex onto the edge.

If *u*≤0/*u*≥1, then the intruding vertex **r** *_k_* is touching one of the capsule's rounded ends and is only interacting with one of the edge's vertices. If 0<*u*<1, then the intruding vertex is touching one of the capsule's sides and is therefore interacting with both edge vertices simultaneously. In this case the interaction force is split between the vertices such that the torque around the contact point is zero and the sum of all forces is also zero:
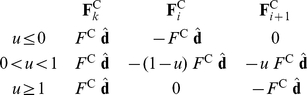
(12)


#### Photophobia

Although cyanobacteria trichomes often display all three types of photomovement, photophobia is considered to be the main driver of pattern formation [12] and therefore only this mechanism is included in the model. The photophobia model is directly based on observations from Gabai [13] who showed that trichomes of *P. uncinatum* are sensitive to step-down reactions at the head as well as step-up reactions at the tail. Photophobic reactions are simulated by reversing the trichome polarity *p* (and thus the trichome movement direction) with probability *q* at each time step. The probability *q* is a function of the light difference between the head and tail of the trichome:
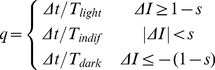
(13)where *ΔI* denotes the difference in average light intensity measured over *c* = 10% of the total trichome length at the trichome head and tail [35], [36]; 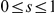 is the photophobic sensitivity of the trichomes and 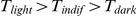 are the average reversal periods for trichomes moving into a lighter area, indifferent light or a darker area. Note that we assume that the light function is normalized such that *I* = 0 corresponds to ‘black’ i.e. total darkness and *I* = 1 corresponds to ‘white’ i.e. the maximum light intensity in the domain. We also assume that *I* = 1 is the optimum light intensity for the trichomes, which they strive to achieve.

#### Simulation setup

**Figure 3:**
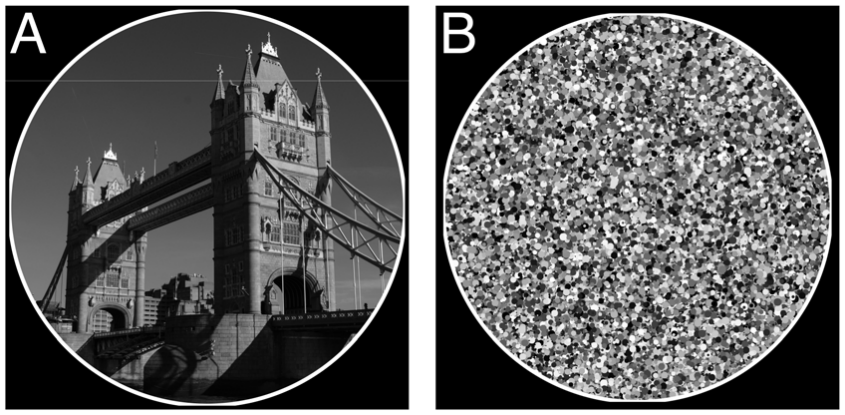
Light fields. (A) Light field used for the first and second round of simulations based on a photograph of the London Tower Bridge. (B) Randomly generated light field used in the third round of simulations. The field is composed of overlapping spots that cover the whole area. Spot diameter and light intensity vary between 0.2–2 mm and 0–1 respectively. Solid white line corresponds to the domain boundary.

For all our simulations a 10 cm disk with variable number of trichomes is initialized such that a certain predefined domain coverage was achieved, as described in the results section. For the static light field a 2024×2024 pixel monochrome picture of the Tower Bridge, London was used (inverted to simulate a photographic negative, Figure 3A). Linear interpolation was used between the pixels to obtain a smooth light field. Each simulation corresponds to either 8 or 16 hours of simulated real time. The default parameter values used are listed in Table 1.

**Table 1:** Parameter values used during simulations.

Type	Parameter	Description	Value		Ref.
Integration	*Δt*	Timestep	0.1	s	
	*η*	Viscosity	1		
Domain	*D*	Diameter	10	cm	
Trichomes	*d*	Diameter	10	µm	[44], [46]
	*L*	Length	0.02–2	mm	
	*l*	Segment Length	∼50	µm	
	*E*	Stiffness	10		
	*v*	Gliding Speed	0–10	µm.s^−1^	[46]
Photophobia	*c*	Size of head/tail	10%	of *L*	[35], [36]
	*s*	Sensitivity	0–1	–	
	*T_dark_*	Short rev. period	20	s	[13], [47]
	*T_indif_*	Medium rev. period	5	min	
	*T_light_*	Long rev. period	120	min	
Dynamic light		Spot diameter	0.2–2	mm	
	*f*	Frequency	0–2048	s^−1^	
	*I*	Avg light intensity	0.5	−	

#### Programming and data analysis

The model was programmed using C++ and CUDA and run on an nVidia GTX295 graphics processing unit. For each simulation up to 400 system states were saved, including each trichome's average exposure at that time. The visualization was done by binning all the vertices from 20 consecutive states into a 2024×2024 pixel domain. This superposition serves to enhance the cyanobacterial “signal” and compensate for the limited number of trichomes that fit in the 2D domain. The binned data was then transformed using the function 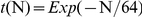 to enhance contract and converted to an 8-bit grayscale image.

## Results

### Modeling cyanographs

As an initial test of our model, we sought to simulate the emergence of cyanographs. Cyanographs are formed by projecting a photographic negative onto a culture of photophobic filamentous cyanobacteria in a Petri dish. Due to photophobic responses, the trichomes redistribute themselves in the light field and after a few hours a photographic positive is formed by the culture. For the cyanograph simulations in this study, a high contrast photograph of the London Tower Bridge (Figure 3A) with a clear sky background forming a vertical gradient was used.

The Petri dish is modeled as a 2D circular domain of 10 cm diameter in which the trichomes are laid. The boundary conditions are rigid and inelastic so that the trichomes are unable to traverse the dish's boundary and are deflected upon collision. Initially, roughly ∼3.5% of the domain is covered with a uniform population of trichomes, all having identical properties. The trichomes are evenly spaced in the domain and each is placed with a random orientation. For the visualization, 20 consecutive states of the simulation are superimposed in order to obtain a clearer “signal” of the cyanobacterial imprint (see Methods).

**Figure 4:**
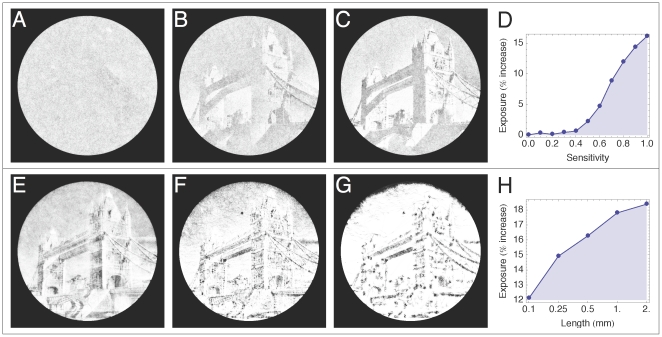
Modeling cyanographs. Images represent 20 superimposed states of a single simulation. Simulations are run with uniform trichome populations. Each parameter value corresponds to a different simulation. Domain radius is 10 cm. The number of trichomes per simulation depends on the trichome length (the coverage of the domain is fixed) and varies between *N* = 1.5×10^4^–2.8×10^5^. Standard errors of the mean (SEMs) were very small due to the large sample sizes and are omitted. (A–C) Length and speed fixed to *L* = 0.5 mm and *v* = 10 µm.s^−1^ respectfully; Sensitivity varied between *s* = 0–1. Images correspond to *s* = {0.4, 0.7, 0.9} and Sup. Movies S1, S2, and S3 respectively. (D) Graph plotting trichome exposure against trichome sensitivity. The photophobic mechanism starts to become effective from s>0.4. Exposure increases roughly linearly, peaking at a 16% increase for “perfectly” sensitive trichomes. (E–G) Sensitivity and speed fixed to *p* = 1 and *v* = 10 m⋅s^−1^ respectfully; length varied between *L* = 0.1–2 mm. Images correspond to *L* = {0.1, 0.5, 2} mm and Sup. Movies S4, S5, and S6 respectively. (H) Log-linear plot of trichome exposure against trichome length (log_2_ scale).

Simulations using different trichome populations were performed. First the photophobic sensitivity parameter (*s*) was varied. This parameter can vary between 0–1, with *s* = 0 representing total insensitivity to light and *s* = 1 “perfect” sensitivity i.e. any difference in light between the trichome ends can trigger a response. Simulations across the entire range of sensitivity were run in *Δs* = 0.1 steps with the trichome length and gliding speed fixed to 0.5 mm and 10 µm.s^−1^ respectively. The results are shown in Figure 4A–D and Sup. Movies S1, S2, and S3. For *s*<0.4 the photographic image does not emerge from the simulations. However for increasing values of *s*>0.4 the picture emerges gradually, similar to a lifting fog, starting with the golden crown at the top of the towers (Figure 4A). For *s*>0.8 the towers become clearly outlined and smaller structures, such as the windows, balconies, stones and buildings on the north bank, begin to reveal themselves in detail (Figure 4B–C). For *s* = 1.0, a dramatic shift in the image quality occurs as a detailed, high contrast picture emerges and one can make out the individual beams, windows, arches and other small substructures which embellish the bridge (Figure 4B). Also, the large shadows cast by the bridge to the left of the picture have disappeared to reveal the structures beneath. The trichomes also move up the gradient in the background, leaving a dark streak at the top of the domain, as well as pick out and enhance small details in the sky, such as the spot between the towers (due to a dirty lens) and the airplane contrails in the upper left corner, which are not present for lower parameter values.

The average steady-state light exposure experienced by the trichomes for each sensitivity value is shown in Figure 4D. Average exposure is measured as the percentual increase above the average light intensity in the light field (approximately 0.72 in the case of the Tower Bridge photograph used). The graph shows that the level of detail shown in the cyanographs correlates positively with increased exposure. Exposure increases significantly with sensitivity, with the most sensitive trichomes receiving 16 percentage points more light than the non-photophobic trichomes.

For the next series of simulations, trichome sensitivity was fixed to *s* = 1 and gliding speed to 10 µm⋅s^−1^ while trichome length was varied using using *L* = {0.1, 0.25, 0.5, 1.0, 2.0 mm}. The results are shown in Figure 4E–H and Sup. Movies S4, S5, and S6. For the shortest trichomes a surprising level of image quality was obtained (Figure 4E). The resulting image displays both detail, with the smaller elements on the bridge clearly visible, and a remarkable level of shading, which is not seen with the longer trichomes. Increasing the trichome length results in decreasing image quality, as the trichomes tend to cluster in small niches of the image and migrate up the light gradient in the sky (Figure 4F). The longest trichomes aggregated into large wave-like clusters in the lightest areas of the photograph and were effective at following the sky gradient towards the top of the domain (Figure 4G).

The average light exposure measured for each population shows that exposure increases with trichome length (Figure 4H), although the effect is less pronounced compared to the sensitivity parameter. Ultimately, the longest trichomes (*L* = 2 mm) distribute themselves such that they receive 7 percentage points more light than the shortest (*L* = 0.1 mm).

### A heterogeneous population in a static light field

**Figure 5:**
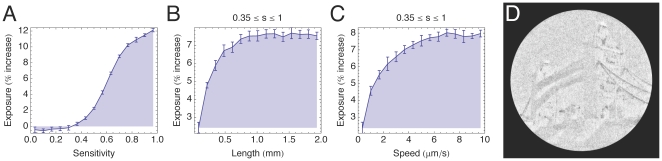
Trichome competition in the Tower Bridge field. Each trichome (*N* = 1.26×10^5^) in the domain is assigned a random length (*L* = 0.02–2 mm), photophobic sensitivity (*s* = 0–1) and gliding speed (*v* = 0–10 µm⋅s^−1^); 11 simulations were performed. Error bars are the standard deviations of simulation means. (A) Graph plotting exposure against photophobic sensitivity. For values *s*<0.35, trichomes are exposed to slightly less than the average light in the domain. For *s*>0.35, exposure increases markedly, peaking at a 12% exposure advantage for “perfectly” sensitive trichomes. (B) Graph plotting exposure against trichome length for sensitivities 0.35<*s*≤1. Exposure increases significantly up to 0.8 mm after which it plateaus. (C) Graph plotting exposure against gliding speed for sensitivities 0.35<*s*≤1. Exposure peaks at 7.5 µm⋅s^−1^ then plateaus. (D) Composite image of 11 simulations after 8 hours.

Next we changed the simulation conditions to see how trichomes with different properties perform when competing for light. For this purpose the same light field was used but with different initial conditions. The domain coverage was increased to 16.2% and each trichome was initialized with a random length (*L* = 0.02–2 mm), sensitivity (*s* = 0–1) and gliding speed (*v* = 0–10 µm.s^-1^). In total 1.2×10^5^ trichomes per simulation were used. The simulation was then run until reaching a steady state and the average exposure of subsets of the population were measured. The results are shown in Figure 5.

In Figure 5A, average exposure is plotted as a function of trichome sensitivity. The trend is similar to what was obtained in the previous simulations, although the least sensitive trichomes are actually slightly less exposed then average. Photophobia takes effect only for sensitivities *s*>0.35, and so we limit our analysis of the effects of trichome length and gliding speed to the parameter range 0.35<*s*≤1.

In Figure 5B, average exposure is plotted as a function of trichome length. Each data point corresponds to a mean over a narrow range of trichome lengths and the full range of sensitivity and gliding speed (which accounts for the drop in average exposure compared to the uniform population simulations). The trend is similar to the results in the previous simulations, except exposure reaches a plateau for lengths *L*>1 mm.

In Figure 5C, average exposure is plotted as a function of trichome gliding speed. Exposure increases with speed peaking at around 6 µm⋅s^−1^ after which exposure stabilizes at 8% above average. The difference in exposure between the slowest trichomes and the fastest is approximately 6 percentage points. Figure 5D shows the resulting cyanograph image when using a mixed population of trichomes.

### A heterogeneous population in a dynamic light field

In microbial mats, trichomes must cope with continuously changing light conditions due to self-shading, sedimentation, changing weather and daylight. To see how the virtual trichomes cope in changing conditions, a procedure for simulating a dynamic light field was devised.

**Figure 6:**
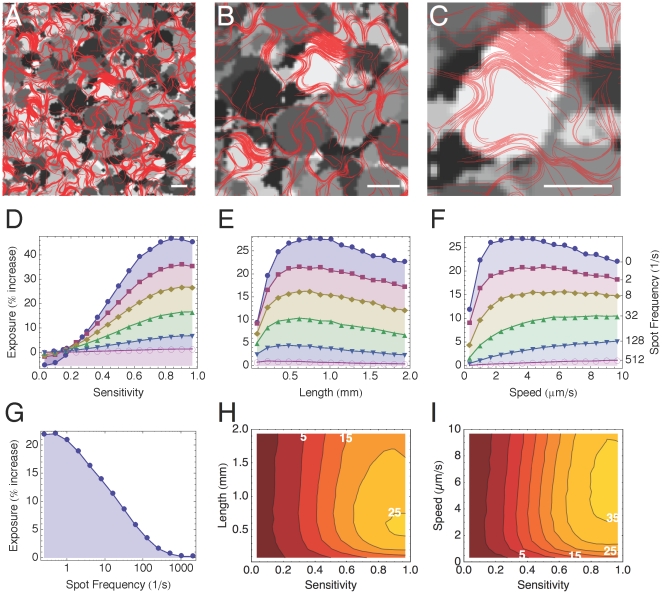
Trichome competition in a dynamic light field. Same initial conditions as in Figure 5, except a light field composed of overlapping circles of different light intensities is used instead of a static photograph. (A–C) Close ups of a simulation displaying the light field (greyscale) and trichomes (red). See Sup. Movie S7. Every 1/*f* sec the light intensity of a random spot in the domain is set to a random value *I* = 0–1. The spots range in diameter between 0.2–2 mm. Scale bars 1 mm. (D–F) Graphs plotting exposure against length (D), sensitivity (E) and gliding speed (F) for six different spot frequencies (0, 2, 8, 32, 128, 512). Different plot colors and symbols correspond to different frequency values, shown on the right-most axis. (G) Plot of average exposure of all trichomes as a function of spot frequency. (H–I) Contour plots demonstrating the relative importance of trichome length vs. sensitivity (H) and gliding speed vs. sensitivity (I). White contour labels indicate percentual exposure increase above average domain light.

First the light field is initialized to uniform intensity (*I* = 0.5). With a given frequency (f), a disk with a random diameter between 0.2–2 mm centred at a random position in the field is filled with a random light intensity between 0–1. Over time the light field becomes a heterogeneous mix of overlapping dark and light disks, although the average intensity remains *I* = 0.5 (Figure 3B). After the field has been completely covered with disks, the trichomes are then placed in the domain as described in the previous section, with the sensitivity, length and gliding speed of each trichome chosen randomly. As the simulation runs, the light field continues to change at the given frequency and the trichomes continuously redistribute to the changing conditions by moving out of darker areas and into lighter ones (Figure 6A–C, Sup. Movie S7).

Simulations were performed over a range of frequencies between *f* = 0–2048 s^−1^. In Figure 6G the average exposure of the entire population of trichomes as a function of the spot frequency is plotted. Exposure decreases with spot frequency as it becomes more difficult for the trichomes to adapt to ever faster changes in the light field.

In Figure 6A–C close-ups of the simulation domains are shown. The overlapping contrasting disks of light and dark of the dynamic light field are in shades of gray and the trichomes are drawn in red. Lighter circles with a high trichome density are more common while darker circles show a lower trichome density, as the trichomes will tend to accumulate in the light traps.

The graphs in Figure 6D–F show the average exposure of the trichomes over a 12-hour period (after the average exposure of the whole population had reached a steady state) as a function of the three parameters *s*, *L* and *v*.

In Figure 6D exposure is measured as a function of sensitivity. The exposure profile here is very similar to what was observed for the cyanograph simulations. Photophobia takes effect starting at a lower sensitivity (*s* = 0.2), which is probably due to the broad range of light intensity of the spots. The least sensitive trichomes, *s*<0.2, are underexposed, receiving less light than the light field mean. Exposure increases rapidly with sensitivity, peaking for trichomes with slightly less than maximal photophobia (*s*∼0.9), which have >40% more exposure than the field mean. Increasing the spot frequency effectively scales the exposure curve downwards, but does not otherwise qualitatively affect the relation.

In Figure 6E, exposure is plotted against trichome length. On average, all the trichomes are able to improve their exposure above the field mean regardless of length, so long as the spot frequency is not too high (*f*<512 s^−1^). For *f* = 0 s^−1^ the light field is static. Unlike the Tower Bridge simulations however, exposure peaks for lengths *L* = 0.61–1 mm and average exposure per trichome decreases slowly for increasing lengths. Increasing the spot frequency effectively scaled the exposure curve downwards. The exposure maximum was always for trichomes of length *L* = 0.61–0.74 mm for *f*<512 s^−1^. This is due to the average diameter of the spots, which is 1.1 mm, whereas the longest trichomes are 2 mm long. The long trichomes manage to accumulate on the smaller bright spots, however their ends protrude from the spot into the darker areas, reducing the average exposure of the trichome (Figure 6C). For higher frequencies the trichomes cannot keep up with the quickly changing light field and are unable to improve their exposure above the light field mean.

In Figure 6F exposure as a function of gliding speed is shown. Exposure rapidly increases with gliding speed, but then peaks after which it declines slowly. The maximum is an increasing function of spot frequency because the faster trichomes cope better with a more quickly changing environment. For a static light field, the best exposure is for obtained *v* ∼3 µm.s^−1^, whereas for spot frequencies *f*≥512 s^−1^ the best exposure is for *v*≥9.5 µm.s^−1^.

In Figure 6H mean exposure across the whole range of spot frequencies tested is plotted against both sensitivity and length in a contour plot. The plot clearly shows the dominant effect of sensitivity over length. For the most sensitive trichomes however, length becomes a significant factor as trichomes in *L*∼0.25–1 mm range do significantly better than longer or shorter trichomes. Finally, in Figure 6I a similar plot combining sensitivity and gliding speed is shown. Again, gliding speed appears to have the strongest effect for highly photophobic trichomes.

## Discussion

Using a cell-based model, we have shown how gliding speed and trichome length complement photophobia and improve the exposure optimization process. Although we have focused on the increasing exposure, the results are also valid for the complementary problem of trichomes seeking to decrease their exposure.

### Photophobia as an exposure optimization mechanism

Using both uniform and mixed populations in static and dynamic light fields, we consistently found that the more photophobically sensitive the trichomes are, the more light they are eventually exposed to. In fact real cyanobacteria have been found to be quite sensitive to light, and differences between head and tail as low as a 4% with a 0.03 lx threshold can trigger a response [12].

Filamentous cyanobacteria have been shown *in vitro* to accumulate in discrete light traps and also distribute themselves in smooth light fields (see Introduction). Although all three types of photomovement can play a role in optimizing light exposure, we found that a simple one instant photophobic mechanism is sufficient to explain the emergence of cyanographs and that this mechanism is also effective in a dynamic light environment, suggesting that photophobia may be an effective strategy *in vivo* (i.e. in microbial mats).

Ramsing and Prufert-Bebourt [37] proposed an alternative strategy. Based on observations of the photomovement of the mat-forming *Microcoleus chthonoplastes*, they found that this organism's strategy is to minimize movement when conditions are good, by either moving less often or increasing the frequency of their reversals. When light is weak, they increase their range of motion, probing the environment for better conditions. They argue that this is a more realistic strategy, as probable conditions within the mat are such that light is diffusive and directionless, making phototaxis untenable, and that the light deeper within the mat varies gradually and that this would make photophobia ineffective. However, as the cyanograph experiments and our simulations show, photophobia can be effective in smooth light fields. It would be interesting to simulate trichomes competing for light using these two alternative strategies and compare.

### Long trichomes enhance exposure optimization

Comparing the distribution of the longest and shortest trichomes in the populations in the Tower Bridge simulations gives some indication as to why the longer trichomes achieve better exposure.

**Figure 7:**
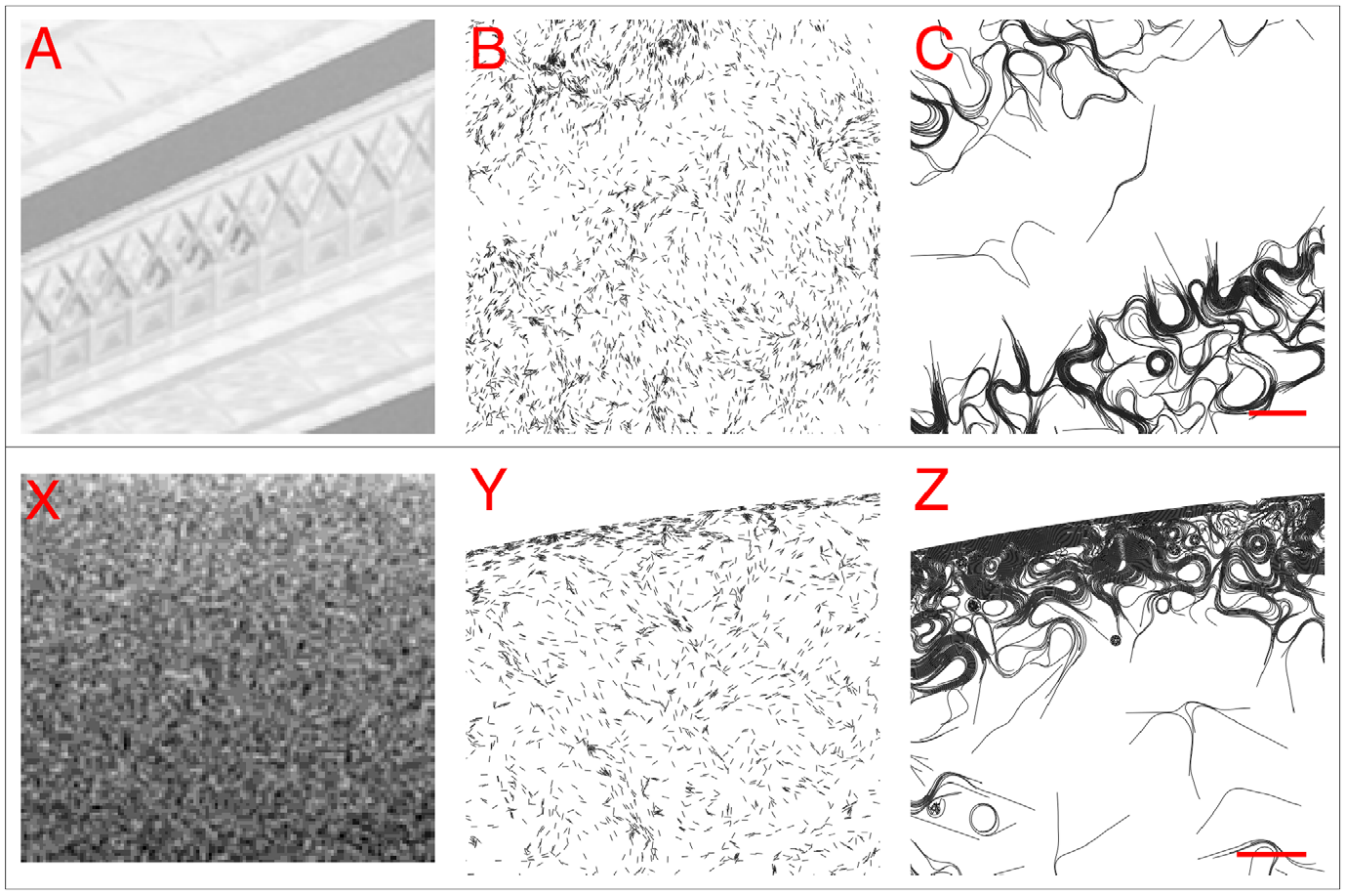
Two portions of the Tower Bridge photographic negative where longer trichomes perform better than shorter trichomes. (A) Close up of the upper cross beams between the towers. (B) The shorter trichomes remain stuck in a finely patterned portion of the crossbeam. (C) In contrast, the longer trichomes are able to find more light below. (X) The photograph background is characterized by a smooth gradient. On the scale of an individual trichome, however, the gradient is weak and noisy. The shorter trichomes are for the most part not able to follow the gradient (Y), whereas the longer trichomes can (Z).

In the first example, the trichomes are exposed to parallel bands of light of varying quality and intensity (Figure 7A). The shortest trichomes are for the most part able to escape the roughly uniform low-intensity bands (Figure 7B), but, unlike the longer trichomes, are unable to cross the adjoining pattern of crosses and concentric boxes in order to reach the better light further down (Figure 7C).

In Figure 7X–Z, we compare the distribution of trichomes at the top of the light domain. This part of the photograph captures the sky on a clear day and is characterized by a smooth light gradient (Figure 7X). In the photographic negative of the image, the sky appears lightest at the top and darkest at the bottom. On the scale of an individual trichome the gradient is fairly noisy and difficult to measure. The shorter trichomes show very little accumulation at the top of the domain, indicating that they are not able to pick up the gradient (Figure 7Y). However, virtually all of the longer trichomes were able to follow the gradient and accumulated extensively at the top of the domain (Figure 7Z).

These examples suggest that the ability of the longer trichomes to measure light between more distant points enhances their capacity to navigate with noisy light signals, detect subtle gradients and avoid falling into small sub-optimal light traps.

Ramsing and Prufert-Bebourt [37] suggested that an effective long-term strategy for the trichomes may be bending into bundles as a way of limiting their motility and compacting themselves in illuminated areas. In our simulations, long trichomes tended to band together and passively curl, forming dense, wave-like sheets as they accumulated in the lighter areas (Figure 6A–C). These structures appeared to be quite stable. Long trichomes that collided with a sheet tended to align with it and added to the structure. Trichomes were only occasionally deflected off of a sheet due to random collisions. Shorter trichomes, however, did not appear to form such structures and were less likely to become bound in this way. We did observe spontaneous alignment of the shorter trichomes, but these swarms were unstable and short lived. The formation of these sheets may be one reason why the longer trichomes perform better in the Tower Bridge simulations, as they may functions as attractors and stabilizers in the brighter areas.

### Fast gliding enhances exposure optimization

Clearly the trichomes have to move in order to distribute themselves effectively over the light field, but if the trichomes move too quickly we have seen that this can actually be detrimental for their overall exposure. One reason for this may be related to the fixed reversal periods used in all the simulations. As these periods are the same for all trichomes, faster trichomes will travel proportionally more between reversal periods. This may cause a trichome to overshoot a small light trap, as it moves past it before a photophobic response can take effect. Reversal periods are likely to be an important parameter for optimizing exposure. Wu et al. [29] showed in their simulations that periodic reversals are essential for efficient Myxobacteria swarming, and that the 8 min average reversal period of these microorganisms is optimal for swarming.

### Is the trichome optimal for photomovement?

What are the advantages of forming trichomes for the cyanobacteria? Trichomes can resemble multicellular organisms due to their impressive degree of cooperation, integration and differentiation. Many filamentous species form differentiated cells within the colony to perform specific functions. Heterocysts specialize in fixing atmospheric nitrogen, which is then distributed to neighbouring cells, whereas akinetes have thickened cells walls and are resistant to cold and desiccation, helping the organism to survive under harsh conditions [38], [39]. Akinetes develop into hormogonia – short and fast trichomes specialized in seeking out better conditions and forming new colonies. *Phormidium uncinatum* (and probably other species) can share power (in the form of proton motive force) across the colony so that only a portion of the trichome need be illuminated for the entire trichome to be energized [40].

Cyanobacterial trichomes are typically only a few micrometers wide and are relatively long, on the order of a few millimetres. Apart from the advantages of colonial life mentioned above, our simulation results indicate that this particular format is beneficial for the trichomes because it helps their photophobic positioning in two ways.

First, our simulations suggest that gliding speeds on the order of a few µm⋅s^−1^ are optimal for photophobic migration, as slower speeds result in worse exposure and faster speeds bring little gain. Filamentous cyanobacteria are able to achieve these speeds, whereas unicellular cyanobacteria typically glide at speeds on the order of a few µm⋅min^−1^. Filamentous species are faster probably due to the streamlined shape of the trichome as well as the coordinated movement of the cells within the colony, whose “slime jet” mechanism can provide a large collective thrust.

Second, we found that long trichomes are also beneficial for optimizing light exposure independently of gliding speed, as they are better at detecting light gradients and are less susceptible to noisy signals. Unfortunately, studies on cyanobacteria rarely include data on trichome length. Studies of *Beggiatoa*, which are not cyanobacteria but are also filamentous and glide, have shown that populations have stable trichome length distributions, with an average length on the order of 1–2 mm and gliding speeds around 3 µm⋅s^−1^ [41], [42]. Since *Beggiatoa* also exhibits phobic and tactic behavior in response to light and oxygen gradients [43], their length and speed may be optimized for these responses.

It is unknown whether trichome length is actively regulated. Typically, trichomes reproduce via transcellular fragmentation. Specific cells in the trichome – the necridia – die and serve as preferable breakage points when mechanical strain is applied by either bending or stretching [42], [44]. There is evidence that the location and timing of necridia formation is under regulatory control, rather than being a totally random event [45]. It would be interesting to associate the regulation of necridia differentiation with population length control, which in turn could be beneficial for phobic and tactic migrations.

#### Inertial vs. non-inertial dynamics and future work

We use plain Newtonian mechanics as the basis for the model dynamics, which includes inertial forces. In reality the cyanobacteria experience no inertia due to their small size and the high viscosity of the slimy environments they inhabit. In the model, the relative importance of inertia versus the propulsion and contact forces is controlled by the mass and viscosity parameters such that the higher the mass and/or viscosity, the less inertia plays a role in the dynamics. Using the generally robust Velocity Verlet numerical integration method for time-stepping the system, we found that we could not set these values arbitrarily high without compromising the stability and accuracy of the numerical method. Although decreasing the time-step improves stability and accuracy, the running time of the simulations increases proportionally. Therefore, the parameter values chosen for the dynamics (timestep, viscosity, mass) reflect a compromise between numerical stability, accuracy and computational efficiency. We found that the dynamics of the system are adequate with the values chosen and observed no oscillations or highly unrealistic behavior.

The model used for this study was conceived to be as simple as possible and is only an initial approach towards implementation of a large-scale cell-based modeling framework suitable for running on GPUs. To improve our framework, we are currently implementing and experimenting with various models of thin elastic rods, mathematical models of photophobia, inertia-less dynamics and higher order integration schemes in full 3D.

Finally, we would like to see the predictions of the model validated through experiments. It should be possible to create cultures with different lengths by using a homogenizer, and trichome gliding speed can be controlled via the agar fraction of the substrate. Trichome sensitivity may be difficult to quantify and control, however the model does not suggest an ideal sensitivity merely that the more sensitive trichomes are to light the better.

## Supporting Information

Movie S1 Gliding, photophobic trichome simulation using a static light field and a homogenous population with low photophobic sensitivity (s = 0.4).
(MOV)

Movie S2 Gliding, photophobic trichome simulation using a static light field and a homogenous population with medium photophobic sensitivity (s = 0.7).
(MOV)

Movie S3 Gliding, photophobic trichome simulation using a static light field and a homogenous population with high photophobic sensitivity (s = 0.9).
(MOV)

Movie S4 Gliding, photophobic trichome simulation using a static light field and a homogenous population of very short trichomes (L = 0.1 mm).
(MOV)

Movie S5 Gliding, photophobic trichome simulation using a static light field and a homogenous population of short trichomes (L = 0.5 mm).
(MOV)

Movie S6 Gliding, photophobic trichome simulation using a static light field and a homogenous population of long trichomes (L = 2 mm).
(MOV)

Movie S7 Gliding, photophobic trichome simulation using a dynamic light field and a heterogeneous population (s = 0–1, L = 0.01–2 mm, v = 0–10 µm.s-1).
(MOV)
